# Investigating diagnostic sequencing techniques for CADASIL diagnosis

**DOI:** 10.1186/s40246-019-0255-x

**Published:** 2020-01-08

**Authors:** P. J. Dunn, N. Maksemous, R. A. Smith, H. G. Sutherland, L. M. Haupt, L. R. Griffiths

**Affiliations:** Genomics Research Centre, Institute of Health and Biomedical Innovation, School of Biomedical Sciences, Queensland University of Technology, Brisbane, QLD Australia

**Keywords:** CADASIL, NOTCH3, Cerebral small vessel disease, Diagnostic testing

## Abstract

Cerebral autosomal dominant arteriopathy with subcortical infarcts and leukoencephalopathy (CADASIL) is a cerebral small vessel disease caused by mutations in the *NOTCH3* gene. Our laboratory has been undertaking genetic diagnostic testing for CADASIL since 1997. Work originally utilised Sanger sequencing methods targeting specific *NOTCH3* exons. More recently, next-generation sequencing (NGS)-based technologies such as a targeted gene panel and whole exome sequencing (WES) have been used for improved genetic diagnostic testing. In this study, data from 680 patient samples was analysed for 764 tests utilising 3 different sequencing technologies. Sanger sequencing was performed for 407 tests, a targeted NGS gene panel which includes *NOTCH3* exonic regions accounted for 354 tests, and WES with targeted analysis was performed for 3 tests. In total, 14.7% of patient samples (*n* = 100/680) were determined to have a mutation. Testing efficacy varied by method, with 10.8% (*n* = 44/407) of tests using Sanger sequencing able to identify mutations, with 15.8% (*n* = 56/354) of tests performed using the NGS custom panel successfully identifying mutations and a likely non-*NOTCH3* pathogenic variant (*n* = 1/3) identified through WES. Further analysis was then performed through stratification of the number of mutations detected at our facility based on the number of exons, level of pathogenicity and the classification of mutations as known or novel. A systematic review of *NOTCH3* mutation testing data from 1997 to 2017 determined the diagnostic rate of pathogenic findings and found the NGS-customised panel increases our ability to identify disease-causing mutations in *NOTCH3*.

## Background

*NOTCH3* (Notch homologue 3) encodes a large single-pass transmembrane receptor that transduces signals between cells [1]. It is highly conserved and critical for cell fate determination in embryonic development, the differentiation and maturation of functional arteries, and the biological processes of tissue injury and repair [1–3]. The expression of *NOTCH3* is ubiquitous in adults; however, due to mutations associated with cerebral autosomal dominant arteriopathy with subcortical infarcts and leukoencephalopathy (CADASIL), some studies suggest that *NOTCH3* also plays a role in maintaining vascular homeostasis [1].

CADASIL is a cerebral small vessel disease affecting the vascular smooth muscle cells (VSCMs) and characterised by *NOTCH3* mutations and/or the presence of granular osmiophilic material (GOM) [4]. The clinical signs and symptoms for CADASIL include recurrent subcortical ischaemic events; cognitive impairment including dementia, migraine, motor disabilities such as gait disturbances, urinary incontinence and pseudobulbar palsy, encephalopathy, mood disturbances such as apathy or severe depression; and less commonly seen neurological manifestations such as seizures [5–7].

*NOTCH3* encodes one of four NOTCH proteins in mammals and is a core component in Notch signalling, which is considered one of the ‘elite’ signalling pathways due to its high conservation across species [8]. The NOTCH3 protein is comprised of distinct structural domains; the extracellular domain (ECD), transmembrane domain and intracellular domain (ICD). The ECD is made up of the epidermal growth factor-like repeats (EGFRs) and LIN12/Notch repeats (LNR), whilst the ICD is made up of the recombining binding protein Janus kinase (RBPJK)-associated module (RAM) domain, ankyrin repeats, nuclear localization signals and a C-terminal PEST (proline, glutamate, serine, threonine) sequence [9]. Each domain has an integral role in Notch signalling including interaction with the EGFRs through ligand binding; the RAM domain physically interacts with an effector protein (e.g. RBPJ or CBF1); the ankyrin repeats mediate different protein-protein interactions, and the PEST domain promotes the degradation of the intracellular domain [10].

In *NOTCH*3 signalling, the ECD of the Notch protein (NECD) binds to a ligand and undergoes a conformational change which exposes a cleavage site for the metalloprotease ADAM17. This change initiates the S-2 cleavage event, through ADAM17, which liberates the ECD from the cell surface [2]. In healthy individuals with no pathogenic *NOTCH3* mutation, the ECD-ligand complex is then removed from the extracellular matrix (ECM) through endocytosis from the ligand-presenting cell, whilst in CADASIL patients, this complex aggregates with other ECM proteins and forms the GOM [2]. Activation of the Notch receptor occurs through an S-3 cleavage event caused by a gamma secretase (e.g. presenilin), which liberates the Notch intracellular domain (NICD) from the cell wall [11]. The NICD either translocates to the nucleus by binding with members of the co-activator complex (e.g. RBP/JK) or interacts with members of other signalling pathways [11, 12].

The result of *NOTCH3* mutations on disease causation is generally due to the location and type of mutation within the gene. CADASIL patients have well-characterised cysteine-altering missense mutations within exons 2–24, which result in the gain or loss of a cysteine residue in 1 of the 34 EGFRs [4, 13–15]. In comparison, truncating *NOTCH3* mutations within exon 33 (often deletions of stop-loss mutations) which disrupts the *NOTCH3* PEST domain are also known to cause lateral meningocele syndrome (LMS) MIM#130720 [16, 17]. The disruption of the PEST domain presumably results in an increased half-life of the NICD and, as a result, prolonged NOTCH signalling [17]. Interestingly, this does not seem to be the case in CADASIL as *NOTCH3* signalling does not seem to be impaired, despite causative mutations being primarily found in the ECD of the protein [18, 19]. There are also several pathological hallmarks of CADASIL which include profound demyelination and axonal damage, as well as arteriopathy caused by degeneration of vascular smooth muscle cells (VSMCs) in the brain and peripheral organs [20–22]. Damage to VSMCs is also thought to cause progressive thickening of the arteriole walls, fibrosis and luminal narrowing in the medium and small arteries eventually resulting in lacunar infarcts [23, 24].

Originally, CADASIL was diagnosed by the presence of granular osmiophilic material (GOM), which contains the ectodomain of the NOTCH3 protein, identifiable in the walls of small arteries via examination of tissue biopsy using electron or light microscopy [4, 25]. However, sequencing of *NOTCH3* is now used as a diagnostic tool with studies finding congruence between *NOTCH3* mutations and GOM in the diagnosis of CADASIL [26, 27]. Where patients have no known identifiable *NOTCH3* mutation, they can also be categorised as being CADASIL-like and if a genetic cause is found could be re-classified as a similar condition (e.g. *HTRA1* mutations in cerebral autosomal recessive arteriopathy with subcortical infarcts and leukoencephalopathy (CARASIL) or *GLA* mutations in Fabry disease) [28, 29]. The Genomics Research Centre (GRC) currently undertakes diagnostic testing for familial hemiplegic migraine, epilepsy, CADASIL, episodic ataxia type 2 and spinocerebellar ataxia type 6, utilising Sanger sequencing, as well as a next-generation sequencing (NGS) 5-gene custom panel (*CACNA1A*, *ATP1A2*, *SCN1A*, *NOTCH3* and *KCNK18*). The GRC also undertakes clinical whole exome sequencing (WES) to diagnose conditions with similar phenotypes to those that can be diagnosed using the NGS 5-gene panel [30]. The aim of this study was to analyse the number and types of mutations identified in CADASIL in referred patients across the three different sequencing techniques.

## Results

**Fig. 1:**
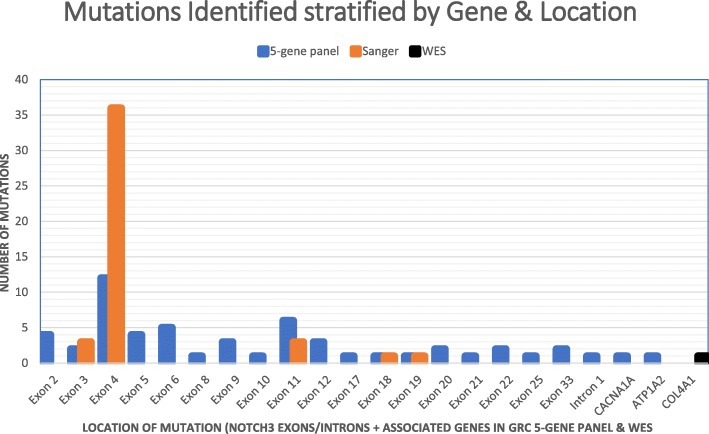
Bar graph stratifies the number of mutations identified in the NOTCH3 exons and introns, CACNA1A and ATP1A2 from using Sanger sequencing (orange bars), the NGS 5-gene panel (blue) and the mutation identified in COL4A1 by whole exome sequencing (WES) in black

Sanger sequencing for *NOTCH3* identified potential causal mutations in 10.8% (*n* = 44/407) of tests performed (Table 1). All potential disease-causing mutations were identified to be heterozygous with mutations located in exon 4 (*n* = 36), exon 3 (*n* = 3), exon 11 (*n* = 3), exon 18 (*n* = 1) and exon 19 (*n* = 1) (Fig. 1 and Table 2). All mutations (*n* = 44) identified by Sanger sequencing in our cohort had previously been reported in the literature, HGMD or dbSNP (Table 2). Interestingly, three samples with Cys-sparing mutations have all been previously identified in CADASIL patients in HGMD and dbSNP (Table 1).

**Table 1:** The number of potential causal mutations identified by the two different sequencing techniques and stratified according to gender (M, male; F, female). *There is an overlap of samples completing multiple sequencing when there has been no mutation identified via the previous sequencing technique which shows an improved diagnostic rate using the GRC NGS 5-gene panel compared to targeted exon Sanger sequencing

Sequencing technique	Sample number tested	Gender and age of testing, ± SD	Mutations identified	Number. of Cys-sparing mutations	Number of pathogenic (HGMD/ClinVar)	Number of unreported mutations
Sanger	M = 139	M = 49.77 ± 13.55	M = 16 (11.5%)	M = 1 (6.3%)	M = 16 (100%)	M = 0 (0%)
	F = 268	F = 50.91 ± 14.12	F = 28 (10.4%)	F = 2 (7.1%)	F = 27 (96.4%)	F = 1 (3.6%)
	M + F = 407	M + F = 50.52 ± 13.94	M + F = 44 (10.6%)	M + F = 3 (6.8%)	M + F = 43 (97.7%)	M + F = 1 (2.3%)
GRC NGS 5-gene custom panel	M = 133	M = 51.60 ± 13.90	M = 25 (18.8%)	M = 9 (36.0%)	M = 15 (60.0%)	M = 14 (56%)
	F = 221	F = 51.01 ± 14.70	F = 31 (14.0%)	F = 12 (38.7%)	F = 23 (74.2%)	F = 8 (25.8%)
	M + F = 354	M + F = 51.39 ± 14.41	M + F = 56 (15.8%)	M + F = 21 (37.5%)	M + F = 38 (67.9%)	M + F = 18 (32.1%)
Total*	M = 244	M = 51.04 ± 13.84	M = 41 (16.8%)	M = 10 (24.4%)	M = 31 (75.6%)	M = 14 (56%)
	F = 436	F = 51.98 ± 14.29	F = 59 (13.5%)	F = 14 (23.7%)	F = 50 (84.7%)	F = 8 (25.8%)
	M + F = 680	M + F = 51.64 ± 14.14	M + F = 100 (14.7%)	M + F = 24 (24%)	M + F = 81 (81.0%)	M + F = 19 (19.0%)

**Table 2:** Potential disease causing variants identified by Sanger sequencing stratified according to exon number and the number of samples with that variant

Mutation	No. of samples	Exon	Cysteine altering	rs number	Previously identified
p.Arg110Cys	2	3	Y	–	HGMD CM971056
p.Arg110Tyr	1	3	N	–	HGMD CM971056
p.Arg141Cys	14	4	Y	–	HGMD CM971058
p.Arg153Cys	6	4	Y	rs797045014	HGMD CM971060
p.Arg169Cys	1	4	Y	rs28933696	HGMD CM961043
p.Arg182Cys	8	4	Y	rs28933697	HGMD CM961044
p.Cys144Phe	3	4	Y	–	HGMD CM001266/HGMD CM001267/HGMD CM003947
p.Cys174Arg	2	4	Y	–	HGMD CM033795
p.His170Arg	2	4	N	rs147373451	HGMD CM107598
p.Arg544Cys	1	11	Y	rs201118034	HGMD CM994179
p.Arg607Cys	1	11	Y	–	HGMD CM003019
p.Cys579Arg	1	11	Y	–	HGMD CM121680
p.Cys573Gly	1	18	Y	–	HGMD HM050017
p.Arg1031Cys	1	19	Y	–	HGMD CM971070

The NGS 5-gene custom panel identified mutations in 15.8% (*n* = 56/354) of patients screened for CADASIL across *NOTCH3* (*n* = 53/56), *CACNA1A* (*n* = 2/56) and *ATP1A2* (*n* = 1/56). This included 52 samples which had previously been tested by Sanger sequencing and where no causative mutations had been identified. The increased diagnostic rate in the samples was also identified to be statistically significant (*p* value = 0.027) by a direction *χ*^2^ analysis based on the hypothesis that the NGS 5-gene panel diagnostic rate will be greater than the Sanger sequencing diagnostic rate. Variants in exons 2–24 of *NOTCH3* accounted for 92.45% (*n* = 49/53) of *NOTCH3* mutations that have been reported in patients (Table 3). The remaining 3 *NOTCH3* variants were identified in exon 25 (p.Leu1518Met) and exon 33 (p.Glu2268Lys) and a deletion in intron 1 (part of the 5′ UTR sequenced from the panel). As the missense mutation in exon 33 does not result in a truncated protein that would disrupt the PEST region and the patient was not identified to have an LMS phenotype, it was considered unlikely that this variant is causative of LMS. In addition, 3 heterozygous missense mutations in other genes within the panel were identified (*CACNA1A*—p.Asp1723Asn and p.Ala987Ser; *ATP1A2*—p.Glu219Gln) suggesting that these patients have familial hemiplegic migraine (FHM) which has symptomatic features which overlap with CADASIL. Our analysis identified known HGMD disease-causing mutations in (*n* = 38/56) of tests (Table 3). *NOTCH3* Cys-sparing mutations accounted for 11.1% (*n* = 5) of mutations identified, all within exons 2–24 (Tables 1 and 3). In addition, there were 3 commonly identified amino acid changing mutations which accounted for *n* = 35/100 total variants (Table 1), including Arg141Cys, Arg153Cys and Arg182Cys, which were identified in 16, 9 and 10 cases, respectively (Tables 2 and 3). All samples with the same mutation were followed-up to check for related family members; however, there was no definitive evidence to suggest a relationship based on the clinical information received upon genetic testing request. However, due to the high number of samples with the same mutation, it is likely given the rare nature of CADASIL that there may be some familial relationship.

**Table 3:** Mutations identified in the GRC Custom 5-gene panel stratified according to the exon, number of samples and, in some cases, the different genes (NOTCH3, CACNA1A and ATP1A2)

Mutation	No. of samples	Cysteine altering	Exon/intron	Between exons 2 and 24	rs number	HGMD disease causing
p.Arg54Cys	2	Y	2	Y	–	HGMD CM003012
p.Asp45His	1	N	2	Y	rs142031490	–
p.Gly53Ser	1	N	2	Y	–	HGMD CM106869
p.Arg113Ter	1	N	3	Y	–	–
p.Arg90Cys	1	Y	3	Y	–	HGMD CM971055
p.Arg133Cys	1	Y	4	Y	rs137852642	HGMD CM971057
p.Arg141Cys	2	Y	4	Y	–	HGMD CM971058
p.Arg153Cys	3	Y	4	Y	–	HGMD CM971060
p.Arg169Cys	1	Y	4	Y	rs28933696	HGMD CM961043
p.Arg182Cys	2	Y	4	Y	rs28933697	HGMD CM961044
p.Asp139Val	1	N	4	Y	rs766608781	–
p.Cys183Arg	1	Y	4	Y	–	HGMD CM001270
p.Cys224Tyr	1	Y	4	Y	–	HGMD CM971065
p.Asp239Asn	1	N	5	Y	–	–
p.Cys233Tyr	1	Y	5	Y	–	HGMD CM052273
p.Cys260Arg	2	Y	5	Y	–	HGMD CM095351
p.Arg332Cys	1	Y	6	Y	rs137852641	HGMD CM014070
p.Cys271Tyr	1	Y	6	Y	–	HGMD CM060011
p.Cys291Ser	1	Y	6	Y	–	–
p.Cys318Phe	1	Y	6	Y	–	–
p.Ser299Arg	1	N	6	Y	–	–
p.Arg449Cys	1	Y	8	Y	–	HGMD CM023659
p.Cys473Leu	1	Y	9	Y	–	–
p.Gly490Ala	1	N	9	Y	rs374248747	–
p.Tyr465Cys	1	Y	9	Y	–	HGMD CM035647
p.Thr514Met	1	N	10	Y	–	–
p.Arg544Cys	1	Y	11	Y	rs201118034	HGMD CM994179
p.Arg587Cys;Arg587Cys	1	Y	11	Y	–	HGMD CM061879
p.Arg607His	1	N	11	Y	rs747661515	HGMD CM003019
p.Asp547Gly	1	N	11	Y	–	–
p.Cys597Trp	1	Y	11	Y	–	–
p.Arg640Cys	1	Y	12	Y	–	HGMD CM125168
p.Arg640Cys	1	Y	12	Y	–	HGMD CM125168
p.Val644Asp	1	N	12	Y	rs148046938	–
p.Pro857Leu	1	N	17	Y	–	–
p.Cys977Gly	1	Y	18	Y	–	HGMD CM050017
p.Arg1006Cys	1	Y	19	Y	–	HGMD CM971069
p.Arg1100Leu	1	N	20	Y	–	–
p.Tyr1106Cys	1	Y	20	Y	–	–
p.Cys1119Tyr	1	Y	21	Y	–	–
p.Arg1231Cys	1	Y	22	Y	rs201680145	HGMD CM971071
p.Arg587Ser	1	N	22	Y	–	HGMD CM061879
p.Leu1518Met	1	N	25	N	rs148166997	HGMD CM119551
p.Glu2268Lys	1	N	33	N	–	–
p.Pro2178Ser	1	N	33	N	–	–
chr19:15311579_15311580delinsTA	1	N	Intron 1	N	–	–
p.Ala987Ser (*CACNA1A*)	1	–	–	–	–	–
p.Asp1723Asn (*CACNA1A*)	1	–	–	–	rs368257155	–
p.Glu219Gln (*ATP1A2*)	1	–	–	–	–	–

This work also yielded five previously unreported *NOTCH3* variants (Table 4) identified through either the NGS 5-gene panel or by Sanger sequencing. *n* = 3/5 variants were Cys-altering and located between exons 2 and 24 whilst the other *n* = 2/5 variants identified were Cys-sparing (CAD-390 Thr514Met and CAD-640 Pro857Leu) also located within exons 2–24. In silico tools for determining pathogenicity, MutationTaster, PredictSNP2, CADD, DANN, FATHMM, FunSeq2 and GWAVA, identified multiple lines of computational evidence which support a deleterious effect on the gene/gene product, whilst *n* = 3/5 variants had only one line of computational evidence which suggested a neutral or non-deleterious effect (CAD-390, CADD; CAD-630, FATHMM; CAD-640, GWAVA). Multiple samples also had an unknown effect for pathogenicity measured by GWAVA (CAD-390, CAD-528 and CAD-535).

**Table 4:** Novel variants identified via Sanger sequencing and the GRC 5-gene panel with in silico predictive scores of pathogenicity including MutationTaster, PredictSNP2, CADD, DANN, FATHMM, FunSeq2 and GWAVA

SAMPLE	Mutation	Cysteine altering	Exon	Between exons 2 and 24	MutationTaster	PredictSNP2 (%)	CADD (%)	DANN (%)	FATHMM (%)	FunSeq2 (%)	GWAVA (%)
CAD-390	Thr514Met	N	10	Y	Disease causing	87	65 (N)	73	56	62	?
CAD-528	Cys291Ser	Y	6	Y	Disease causing	87	84	60	83	62	?
CAD-535	Cys318Phe	Y	6	Y	Disease causing	87	84	60	82	62	?
CAD-630	Cys473Leu	Y	9	Y	Disease causing	82	84	77	63 (N)	62	51
CAD-640	Pro857Leu	N	17	Y	Disease causing	87	80	77	72	62	53 (N)

The percentage indicates how confident the tool is for determining a deleterious, neutral (N) or unknown (?) variant effect. Percentages listed with “(N)” indicated the percentage of confidence in calling a benign or non-damaging variants based off the in silico tool used. “?” indicates that the in silico tool could not determine whether the variant would be pathogenic or damaging

In the study data set, there were three samples which were previously tested using the NGS 5-gene panel that had WES only completed with targeted analysis on *NOTCH3* as well as *COL4A1* and other specified genes. All samples had previously been tested using the NGS gene panel, and no potential causative mutation had been identified. Of these, one sample was identified to have a variant of unknown significance in *COL4A1* (p.Gly1198Arg) which was predicted to be pathogenic by in silico tools such as SIFT, PolyPhen and MutationTaster*.* There were no other clinically significant variants identified in other genes requested for analysis known to cause related CSVDs including *HTRA1*, *HTRA4*, *COL4A1*, *COL4A2*, *ARX*, *TREX*, *GLA* and *NOTCH3* in CAD-661, and *NOTCH3*, *APP*, *COL4A1*, *COL4A2*, *TREX1*, *ARX*, *HTRA1*, *HTRA2*, *GLA* or *ITM2B* in CAD-637. *NOTCH3* was analysed for all three samples by WES and found to confirm 100% concordance with the NGS gene panel results for variants identified.

## Discussion

Sequencing of *NOTCH3* is a critical component in the diagnosis of CADASIL. Initial diagnostic testing for *NOTCH3* mutations was influenced by research conducted by Joutel et al. [31] and subsequent supporting literature which identified mutations clustering within exons 3 and 4 of the gene [15, 32]. It is partially due to this that there remains a bias in mutations detected via Sanger sequencing in exon 4 due to the initial primary sequencing of *NOTCH3* being limited to exons 3 and 4. The GRC NGS 5-gene custom panel data also supports the clustering of mutations in exon 4; however, there is a greater spread of mutations across all *NOTCH3* exons, with most of the identified mutations found within exons 2–24 [33].

The development and design of the NGS 5-gene panel in 2012 was completed as it allowed for a cost- and time-effective approach to identify mutations in any of the 33 *NOTCH3* exons as opposed to individual exons sequenced at an increased cost if no mutation is initially identified [30, 34]. The ability of the custom panel to sequence all exons and flanking untranslated regions has led to an increased diagnostic rate, from 10.6 to 15.8% (*p* value = 0.027) (Table 1) and can include identifying previously unreported variants (Table 4). Whilst the majority of mutations identified through the gene panel were Cys-changing and located between exons 2 and 24, a number of variants were identified which do not disrupt the cysteine residues in EGFR. Cys-sparing mutations are contradictory to the hypothesis that Cys-changing mutations in *NOTCH3* are responsible for the disease mechanism in CADASIL; however, multiple case studies have identified Cys-sparing mutations in *NOTCH3* (p.R61W, p.R75P, p.R213K, p.A1020P and p.T1098S) as a cause of CADASIL [35–40]. Other studies have also identified mutations located outside the EGFRs implicated as the cause for CADASIL and white matter disease, suggesting that there are other mechanisms which contribute or cause the CADASIL phenotype [41, 42]. The increase in mutations that do not affect Cysteine residues or the EGFRs are reflected in updated proposed guidelines for CADASIL diagnosis which suggest that non-Cysteine-altering mutations should also be investigated carefully [43, 44].

Variants identified in other genes in the panel (Table 3) were due to clinical requesting for further analysis on patients with no identifiable *NOTCH3* mutation. This was seen with mutations identified in *CACNA1A*, *ATP1A2* and *COL4A1*. Mutations in *CACNA1A* are known to cause familial hemiplegic migraine type 1 (FHM1) and episodic ataxia type 2 (EA2). The clinical signs of FHM1 overlap significantly with CADASIL, with migraine reported in ~ 20–35% of CADASIL patients and some motor effects may resemble stroke effects [45, 46]. Due to a lack of prior clinical information, we cannot exclude other aetiologies for the ischaemic events, e.g. if they are due to environmental or lifestyle stresses, vasoconstrictive drugs used as a prior treatment or if another gene mutation not tested is the cause [45, 47, 48]. Another heterozygous gene mutation was identified in *ATP1A2* in CAD-400 that is known to cause familial hemiplegic migraine type 2 (FHM2) (MIM#602481). A meta-analysis completed by Cole and Kittner [49] found an association of greater risk for ischaemic stroke in migraine sufferers. Studies by Harriott et al. [50] failed to reproduce results when investigating *ATP1A2* polymorphisms and stroke risk; however, they did concede that the data from the study is hypothesis-generating and further studies may be useful.

WES identified a heterozygous mutation in *COL4A1*, which is known to cause a cerebral small vessel disease (SVD) with symptoms including transient ischaemic attacks, adult-onset haemorrhagic stroke, periventricular brain abnormalities, white matter hyperintensities and leukoencephalopathy (including cerebellar hypoplasia, cerebral atrophy and vascular changes) [51–53]. Choi [54] highlighted phenotypic similarities between *COL4A1* SVD and *NOTCH3* mutations in CADASIL, showing that both conditions cause lacunar infarcts, cognitive deficits, intracerebral haemorrhage and migraine. The main pathological finding difference involves a defect in the basement membrane as opposed to the GOM found in the arteriole walls, which is difficult to determine unless a tissue biopsy is performed [4, 54].

Despite the limited number of samples assessed in this study, we already have evidence that the use of WES can expand our capabilities to identify genetic causes of cerebral small vessel disease when CADASIL mutation testing is negative. We are also confident that this work is able to identify variants consistently across the different sequencing technologies as stringent validation of this work has been completed for accreditation for diagnostic testing through the National Association for Testing Authorities (NATA), Australia, and through previous work completed by Maksemous et al. [30]. However, one of the limitations in using WES for CADASIL-related conditions is the reliance on the clinician to request the genes for analysis and the potential non-specific symptoms of patients. It is important to identify the correct causative genetic mutation in CADASIL and related conditions as physicians need to be able to manage the symptoms of these disorders. One example related to a major CADASIL symptom is that migraine treatments should include non-steroidal anti-inflammatories (NSAIDs) or analgesics, whilst vasoconstrictors should be avoided due to an increased risk of inducing an ischaemic event [6]. This highlights the need to have open communication between the referring clinicians and the diagnostic testing facilities to ensure gene lists are ready for use in cases where further testing may be required as it can have direct treatment/management ramifications for people affected. Furthermore, detailed phenotypic information is essential to augment the clinical and genetic testing information for improved diagnosis and reporting.

## Conclusions

The role of *NOTCH3* testing in CADASIL diagnosis is important, and with advances in sequencing technology (from Sanger sequencing to NGS gene panels, WES and whole genome sequencing), we can continue to improve diagnostic success rates. However, the number of mutations we are able to identify in patients which are thought to be symptomatic is still quite low. This may be related to limitations associated with the gene panel caused by the small gap in coverage in exon 24 of *NOTCH3*; however, this limitation is unlikely to have a large impact as the coverage gap size and location are not known hotspots for *NOTCH3* mutations in CADASIL. Other genetic mutations known to be associated with similar clinically presenting diseases (FHM1 in *CACNA1A*, FHM2 in *ATP1A2*, and mutations within *COL4A1* cause *COL4A1*-associated leukoencephalopathy) have been identified through follow-up testing requested by clinicians. This supports the premise that the cause of the symptoms of CADASIL may be attributed to other related neurological disorders with overlapping symptoms. The development and implementation of the GRC NGS 5-gene custom panel have shown complete concordance with Sanger sequencing but extends our capacity to detect mutations and resulted in an increased diagnostic rate of 10.8 to ~ 15.8%. Hence, NGS has increased our capabilities to identify *NOTCH3* mutations causative of CADASIL, although the increased variety and relatively low diagnostic yield highlight that there may be other genes or mechanisms which contribute to or cause CADASIL. Future WES and whole genome sequencing may play an important role in identifying other genes implicated in this disorder.

## Materials and methods

Patients were originally referred to the Genomics Research Centre NATA (National Association of Testing Authorities, Australia)-accredited diagnostic laboratory by physicians in Australia and New Zealand. Ethical approval for these studies is through QUT HREC (Approval Number 1400000748). Patient results were selected from internal de-identified records from January 1, 1997, to December 31, 2017, and were based on referrals for CADASIL or CADASIL-like symptoms and specific *NOTCH3* testing. The results were excluded if the samples were identified to have also been sent for testing for familial hemiplegic migraine or if they were family members of previously investigated probands, investigated or used for confirmatory testing based on previous genetic testing for CADASIL. The results were stratified through the identification, exon location and mutation type within *NOTCH3.*

Requested CADASIL/*NOTCH3* patients (*n* = 407) underwent initial Sanger sequencing on exons 3 and 4, unless another exon or an extended *NOTCH3* analysis (sequencing of exons 2, 11, 18 and 19) was subsequently requested. All exons were initially selected for analysis and were based on mutational hotspots identified in *NOTCH3* by Joutel et al. and Peters et al. [15, 27, 32]. The primer sets were designed to encompass some of the entire exon examined, as well as surrounding intronic material, spanning in size from 193 bp for exon 2, 296 bp for exon 3, 488 bp for exon 4, 367 bp for exon 11, 258 bp for exon 18 and 350 bp for exon 19. The methods used for *NOTCH3* Sanger sequencing has been previously described by Roy et al. [55]. Genomic DNA was extracted from peripheral blood lymphocytes using the QIAGEN QIAcube™ (Venlo, Netherlands). Samples were originally sequenced using Sanger et al. [56] dideoxy methods using the ThermoFisher BigDye™ Terminator v3.1 Cycle Sequencing Kit (Thermo Fisher Scientific, Scoresby, Victoria, Australia) and were analysed following separation on an Applied Biosystems™ 310, 3130 or 3500 Series Genetic Analyzer (Thermo Fisher Scientific, Scoresby, Victoria, Australia) [55].

The NGS panel sequencing was designed by Maksemous et al. [30] and provides sequencing information on 92.79% (8071 bp) of *NOTCH3* including the 3′ and 5′ untranslated regions (UTRs). The missing region includes 175 bp in exon 1 (hg 19, chr19:15311617-15311792) and a 407-bp region in exon 24 (hg19, chr19:15288427-15288834). Library preparation was performed using the Ion AmpliSeq library kit 2.0 (Thermo Fisher Scientific, Scoresby, Victoria, Australia) according to the standard protocol (Cat. no. 4480441, Rev. 4.0) with template preparation performed on the Ion PGM OT2 200 Template Kit (Thermo Fisher Scientific, Scoresby, Victoria, Australia), according to the manufacturers’ instructions (part no. 4480974 Rev. 4.0) [30]. Sequencing was performed on the Ion Torrent Personal Genome Machine (PGM) system using Ion Sequencing 200 Kit V2 and an Ion 316 Chip (Thermo Fisher Scientific, Scoresby, Victoria, Australia) according to the manufacturers’ procedures (Cat. no.4482006 Rev.1.0) [30]. Pearson’s chi-square test was also completed based on the hypothesis that there is a greater percentage of mutations identified by the NGS panel compared to Sanger sequencing.

Whole exome sequencing (WES) was performed using Ion AmpliSeq™ Exome Library Kit Plus (Carlsbad, CA, USA) according to the manufacturers’ instructions (MAN0010084). Template preparation, enrichment and chip loading were performed using the Ion PI™ Hi-Q™ Chef Kit (Catalogue number A27198) on the Applied Biosystems Ion Chef (Carlsbad, CA, USA). Sequencing was undertaken on the Ion Proton™ platform (Carlsbad, CA, USA). Only requested genes by physicians were analysed in the WES data, and these included amyloid beta precursor protein (*APP*), aristaless-related homeobox (*ARX*), collagen type IV alpha 1 chain (*COL4A1*), collagen type IV alpha 2 chain (*COL4A2*), high-temperature requirement A serine peptidase 1 (*HTRA1*), high-temperature requirement A serine peptidase 2 (*HTRA2*), high-temperature requirement A serine peptidase 4 (*HTRA4*), three prime repair exonuclease 1 (*TREX1*), galactosidase alpha (*GLA*), *NOTCH3* and integral membrane protein 2B (*ITM2B*) although not all of these genes were investigated in each patient sample*.*

Variant annotation for the NGS techniques was based on the use of population databases and in silico prediction tools for determining pathogenicity. Population databases used for analysis include 1000 Genomes (1000G), exome aggregation consortium database (ExAC) http://exac.broadinstitute.org and genome aggregation database (GnomAD) http://gnomad.broadinstitute.org/. In silico prediction tools used included SIFT (score < 0.05), PolyPhen (score > 0.8), MutationTaster and PredictSNP2 (which also includes CADD, DANN, FATHMM, FunSeq2 and GWAVA [41, 57–59]. Other databases for investigating variant effects included dbSNP https://www.ncbi.nlm.nih.gov/snp/, HGMD http://www.hgmd.cf.ac.uk/ac/index.php and OMIM https://www.omim.org/.

## Data Availability

All data relevant for this study is included within this manuscript; any further information may be made available on request.

## Abbreviations

CADASIL: Cerebral autosomal dominant arteriopathy with subcortical infarcts and leukoencephalopathy
CARASIL: Cerebral autosomal recessive arteriopathy with subcortical infarcts and leukoencephalopathy
ECD: Extracellular domain
EGFR: Epidermal growth factor-like repeats
FHM: Familial hemiplegic migraine
GOM: Granular osmiophilic material
GRC: Genomics Research Centre
HGMD: Human Gene Mutation Database
ICD: Intracellular domain
LMS: Lateral meningocele syndrome
LNR: LIN12/Notch repeats
NATA: National Association of Testing Authorities, Australia
NECD: Notch extracellular domain
NGS: Next-generation sequencing
NSAIDs: Non-steroidal anti-inflammatories
PEST: Proline glutamate, serine, threonine (amino acid) domain
RAM: RBPK-associated module
RBPJK: Recombining binding protein Janus-kinase
UTR: Untranslated region
VSMCs: Vascular smooth muscle cells
WES: Whole exome sequencing
